# Editorial: Early Life Origins of Immune-Mediated Disease

**DOI:** 10.3389/fimmu.2020.02050

**Published:** 2020-09-03

**Authors:** Chrysanthi Skevaki, Catherine A. Thornton

**Affiliations:** ^1^Institute of Laboratory Medicine, Universities of Giessen and Marburg Lung Center (UGMLC), Philipps University Marburg, German Center for Lung Research (DZL) Marburg, Marburg, Germany; ^2^Institute of Life Science, Swansea University Medical School, Swansea, United Kingdom

**Keywords:** pregnancy, infancy, microbiome, breast milk, fetal programming

The transition from *in utero* to *ex utero* immunity is a gradual process, in which the tolerant fetal immune milieu wanes as postnatal immunity develops and this period may be a critical window for the development of immune-mediated disease. Deficiencies in adaptive immune responses in early life are known to contribute to enhanced morbidity and mortality during this time period. However, much less is known about postnatal innate immune ontogeny in health and disease especially at mucosal surfaces, or the genetic factors and environmental exposures that could potentially modulate this developmental trajectory. In this special topic exploring current paradigms in immune programming and the inflammatory response in particular, Georgountzou and Papadopoulos offer an extensive state-of-the-art overview on postnatal development of barrier function, innate immune cells, complement, and cytokines along with associated immune pathways. Reference is made to factors shown to shape innate immune function while at the same time linked to chronic inflammatory disorders such as respiratory allergies. Future research is required to highlight windows of opportunity with appropriate interventions to reduce long term morbidity.

Neonates, particularly when born preterm are more susceptible to infections and sepsis, which has been attributed to the relative immaturity of their immune response to pathogens. Early life exposure to infections though, may lead to reduced risk for late onset sepsis, and perinatal inflammation has been linked to decreased incidence of asthma and atopy later in life. Along the same lines, prolonged empiric administration of broad-spectrum antibiotics in the absence of documented infection is associated with increased risk for late-onset sepsis and death. Vaccinations during the first month of life on the other hand, have not offered significant protection with the exception of tuberculosis and hepatitis B. The impact of early life infections, antibiotics, and vaccinations are discussed in a holistic approach in the exciting review by Raymond et al.. The authors also propose modes for potential immunomodulation and training of the neonatal immune system.

Recently, the concept of an inherent immune deficiency among infants has been challenged. Dantoft et al. investigated the response to human cytomegalovirus infection (HCMV) and found comparable qualitative and quantitative responses between adult and neonatal dendritic cells, albeit with a delay of neonatal cells in regards to interferon subnetworks. Collectively, their findings point to a set-point control mechanism rather than immaturity, which may also better explain the resilience of neonates to infection despite their susceptibility.

One of the most well-documented risk factors for the development of asthma are severe viral (mainly rhinovirus-RV and respiratory syncytial virus-RSV) lower respiratory tract infections in the first years of life. Susceptibility to virus-induced bronchiolitis is influenced by factors such as genetic predisposition, early age and atopy, which in turn shape the host immune response to these viruses. Lynch et al. discuss the role of microbiota composition and diversity in susceptibility to virus-associated wheezing, via their effects on immune system homeostasis and respiratory disease. The authors propose that culprit perturbations in the maternal and infant microbiota could be targeted in the future as a strategy for primary prevention of asthma.

Microbiota contributions and nutrition especially that related to breast feeding and breast milk are two areas of particular debate in regulating immune maturation and risk of immune-mediated disease from early life. Yasmin et al. investigate the effects of various key environments including antibiotic exposures and formula feeding on gut microbial succession that occurs in later infancy. They quantify longitudinal changes in gut microbiota using 16S high-throughput gene sequencing to highlight the dramatic effects of early cessation of breast feeding on abundance of microbial species. Their findings contribute to our understanding of normal gut microbial development in infancy, in this case from 3 to 12 months of age, and lay foundations for ongoing work to elaborate how deviations contribute to and might predict later disease development. Complementing this work, Xiao et al. provide an overview of the role of bioactive components of breast milk, especially human milk oligosaccharides (HMOS) and their metabolites, in creating a fit and resilient immune system and preventing development of type 1 diabetes in particular. The important contribution of breast milk to maintaining barrier integrity within the gastrointestinal tract especially via ensuring that the regulatory function of tolerogenic dendritic cells is ideal is discussed. Connection to the microbiome when considering the effects of human milk on immune development within the gut is unavoidable and here is made via the bacterial metabolism of HMOS to short chain fatty acids implicated in many key immunoregulatory pathways that are dysregulated in immune-mediated disease. These authors also raise the prospect of using HMOS as an early life intervention to ensure optimal immunomodulatory functions and prevent type 1 diabetes.

Goedicke-Fritz et al. pick up on the theme of windows of opportunity for imprinting metabolism and immunity and discuss the particular risks and challenges of preterm birth. Insights gained from improved understanding of immunomodulatory mechanisms functional in preterm infants and their interaction with intrauterine and extrauterine environments from stress to allergens are noted to offer a unique opportunity to contribute to our understanding of disease mechanisms. Given our emerging appreciation of set-point control mechanisms rather than immune immaturity *per se* in determining the role of immune function in early life in disease susceptibility, as discussed above, then studies of premature infants can contribute unique pieces to the puzzle. The highly controlled care of preterm infants also affords an opportunity to make key measures over prospective follow up to address some of the research and clinical questions raised by the authors.

Continuing on the theme of the potential role of microbiota in the transition from *in utero* to *ex utero* immunity, Van der Leek et al. review the role of tryptophan metabolism in immunoregulatory processes linked to the gut microbiome. Providing a comprehensive overview of tryptophan degradation products and pathway metabolites in neurological and immunoregulatory pathways they develop the evidence linking the kynurenine and indoleamine-2,3-dioxygensase pathway to the development of allergic and other immune-mediated diseases. They highlight the knowledge gaps related to this pathway on regulating Th1/Th2/Th17 balance and regulatory T cell function in early life and draw our attention to this as a reasonable target for prevention strategies.

All of these contributions to the Research Topic have stressed the need to better understand life stage specific—from preterm and throughout infancy—features of metabolism and immunity including both how these are developmentally programmed and the effects of various intrauterine and extrauterine environments. The authors who have contributed have the shared goal of driving innovative approaches to understanding, treating and preventing immune-mediated diseases and recognize the need for interdisciplinary efforts to achieve this.

## Author Contributions

All authors listed have made a substantial, direct and intellectual contribution to the work, and approved it for publication.

## Conflict of Interest

CS: Consultancy and research funding, Hycor Biomedical, Bencard Allergie, and Thermo Fisher Scientific; Research Funding, Mead Johnson Nutrition (MJN). The remaining author declares that the research was conducted in the absence of any commercial or financial relationships that could be construed as a potential conflict of interest.

